# An analysis of working dog exposure to environmental contaminants at the Surfside building collapse

**DOI:** 10.3389/fvets.2026.1725234

**Published:** 2026-03-02

**Authors:** Erin Beth Perry, Michelle Karpinsky, Kelly Bender, Stephen Liang, Eileen K. Jenkins, Dakota Discepolo, Evan T. Marquart, Lauryn DeGreeff

**Affiliations:** 1 Southern Illinois University, Carbondale, IL, United States; 2 Florida International University, Miami, FL, United States; 3 Washington University School of Medicine, St. Louis, MO, United States; 4 Huntsville Veterinary Specialists & Emergency, Huntsville, AL, United States

**Keywords:** chemical exposure, decontamination, disaster, microbiological exposure, working dog

## Abstract

**Introduction:**

Working dogs deployed to disaster settings face exposure risks to environmental contaminants. On June 24, 2021, a 12-story beachfront condominium partially collapsed in Surfside, FL, leading to the deployment of Federal Emergency Management Agency (FEMA) urban search and rescue (US&R) teams including working dogs. Hazards identified at the site during ongoing US&R operations included concrete/silica dust, biological materials, household chemicals, pool chemicals/fuel/other regulated materials (ORM), and products of combustion.

**Methods:**

Samples were analyzed from areas of standing water throughout the search area. Surface wipes were collected from the coat of exposed working dogs as well as reward items utilized by handlers during search operations. Chemical and microbiological contaminant profiles were developed.

**Results:**

Elevated levels of benzene, toluene, ethylbenzene, and xylenes (BTEX) and polycyclic aromatic hydrocarbons (PAH) were identified in all fluid samples. Members from the Pseudomonadaceae and Staphylococcaceae family were detected in samples obtained from the center of the search area.

**Discussion:**

Environmental samples collected directly from search areas where dogs are utilized have not previously been tested. These preliminary data are the first to identify chemical and microbial contaminants working dogs may come in direct contact with during search, rescue and recovery operations after a large building collapse. Generation of chemical and microbiological contaminant profiles can inform handlers of real-world risks to working dogs and better inform decontamination procedures to safeguard working dog and human health.

## Introduction

On June 24, 2021, Champlain Towers South, a 12-story beachfront condominium in Surfside, Florida, partially collapsed. Over 80 rescue units responded to the structural collapse site, including FEMA (Federal Emergency Management Agency) urban search and rescue (US&R) teams. In the days that followed, teams searched for survivors and victims, working in 12-h shifts around the clock (1). At one point, rescue efforts were interrupted due to a fire burning deep within the building rubble. Ultimately, the fire was contained and US&R personnel resumed the search for missing residents. In total, 98 people died in the collapse and many others were injured. Given the extent of the disaster and efforts to locate and recover human remains, search and rescue working dogs factored prominently in the response to this building collapse.

Working dogs deployed to disaster settings are likely to encounter chemical contaminants in the environment (2). Toxicants released from natural and urban disasters such as floods, fires, and building collapses can include polycyclic aromatic hydrocarbons (PAH), polychlorinated biphenyls (PCB), dioxins, and other various organic compounds as well as inorganics including heavy metals and asbestos (3). Following the 9/11 attacks at the World Trade Center as well as the Pentagon, working dogs were deployed and utilized in rescue efforts (4, 5). A study of police canines deployed to the World Trade Center site found evidence of environmental toxin absorption in serum samples (6). Subsequent long-term surveillance of search and rescue canines did not identify any significant long term health effects attributable to their service at 9/11 (7). Nevertheless, real-world data on working dog exposure to chemical contaminants after a building collapse remains limited.

Little information is available regarding microbial exposure following a building collapse. The warm and humid weather that occurred in the Surfside area following the collapse was ideal for the proliferation of pathogens associated with sewage. Compromised sewage handling systems as well as the presence of human and animal remains may contribute to microbial contamination of the search environment. Microbiological exposures leading to waterborne infectious diseases after storms and other hydrologic disasters have been well-described in the human health literature. In fact, a 23-year period analysis of infection occurrence following tropical storms during the Atlantic storm season in the U.S. indicated a 48% increase in shiga toxin-producing *Escherichia coli* infections 1 week after storms and a 42% increase in *Legionella pneumophila* infections up to 2 weeks following the storms (45).

Environmental hazards anticipated by US&R teams responding to the Surfside building collapse included concrete/silica dust, household chemicals, pool chemicals/fuel/ORM, products of combustion, raw sewage, and human and animal remains. Warm, humid weather conditions and intermittent rainfall after the building collapse created areas of standing water throughout the operational site. In this study, we sought to characterize chemical and microbiological contaminants present at the Surfside collapse site during ongoing recovery operations involving US&R working dogs.

## Materials and methods

### Ethics approval

Due to the observational nature of this study, a waiver for IACUC approval was issued by Southern Illinois University (Animal Assurance #D16-00044).

### Sample collection

**Figure 1:**
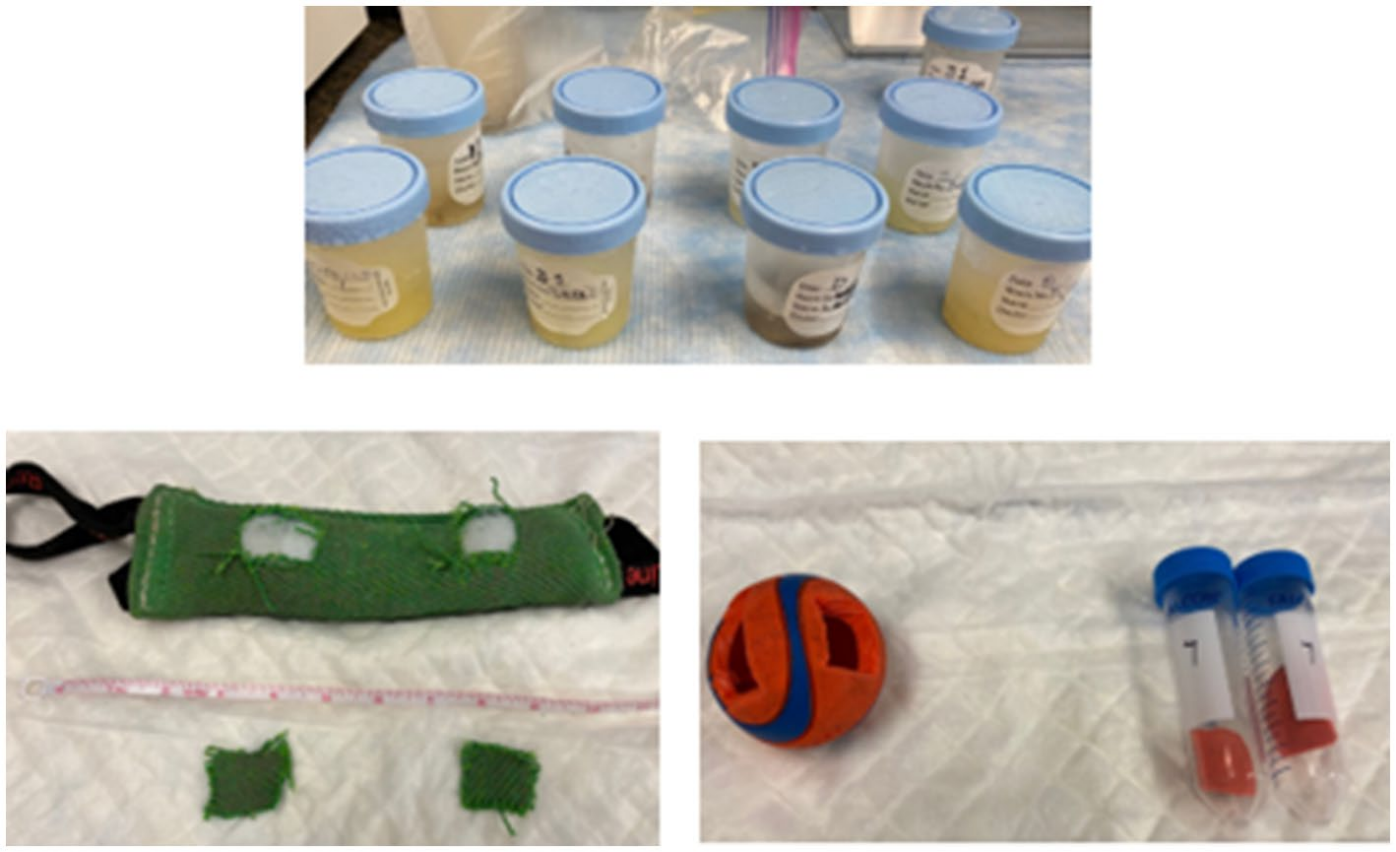
Samples collected from the Surfside building collapse operational site.

Fluid samples were collected from multiple areas of standing water across the Surfside building collapse site (Table 1) approximately 20 days following the building collapse, stored in sterile polypropylene collection tubes on ice (Figure 1), and transported to Southern Illinois University where they were stored at −39° C. Frozen fluid samples were thawed, and aliquots (3 mL) were captured for sub-sampling. Environmental baseline control samples were collected within 30 days of the event from 15 locations in the surrounding area of South Florida. These samples were stored in 60 mL VOA vials at 0° C until analysis.

**Table 1:** Sample inventory including type, source, and description.

Sample	Type	Source	Description
1	Surface wipe	Pre- Canine 1	NA
2	Surface wipe	Pre- Canine 2	NA
3	Surface wipe	Pre- Canine 3	NA
4	Surface wipe	Post- Canine 4	NA
5	Surface wipe	Post- Canine 5	NA
6	Control Gauze	Control	NA
7	Reward Item	Used Ball	After use
8	Reward Item	Used Tug toy	After use
9	Fluid	Environmental	Building 1 Puddle 2
10	Fluid	Environmental	Building 1 Puddle 3
11	Fluid	Environmental	Decontamination Area, East
12	Fluid	Environmental	Decontamination Walkway
13	Fluid	Environmental	Decontamination Area 2 Puddle
14	Fluid	Environmental	Building 2 Puddle
15	Fluid	Environmental	Building 1 Puddle
16	Fluid	Environmental	Parking Lot
17	Fluid	Environmental	Center of a pile of rubble

Surface wipes from five FEMA US&R working dogs using dry sterile gauze were collected immediately following search and recovery operations and within 24 h of fluid sample collection (Table 1). Of those, three dogs were sampled prior to decontamination and two were sampled in the same manner following decontamination (conducted by the individual handlers). Dogs were allowed to remain in a position of comfort (either stand or sit) while the gauze wipe was applied with gentle pressure directly to the dorsal aspect of the dog from the shoulder to the hip by a single trained researcher using clean nitrile gloves. An unused wipe was also collected to serve as a control. The wipes were placed into sterile polypropylene tubes on ice and shipped overnight to Southern Illinois University where they were stored at −39 °C.

Two reward items (tug toy and rubber ball) used for dogs within the search site were also submitted for analysis. Those items were used by handlers during operations, are commonly held in the working dog’s mouth and are often in direct contact with environmental contaminants.

Liquid aliquots, gauze wipe segments (2″ x 2″) and reward item segments (2″ x 2″) were shipped overnight on chemical ice packs to Florida International University and placed into storage at 0° C upon receipt pending additional analyses.

### Chemical analysis

Chemical profiles were completed for both targeted and untargeted compounds in environmental fluid, surface wipe, and reward item samples. Targeted chemical analysis focused on three classes of compounds: benzene, toluene, ethylbenzene, and xylenes (BTEX) polycyclic aromatic hydrocarbons (PAH) and polychlorinated biphenyls (PCB). The AROMA-VOC (Entanglement Technologies Inc.) trace vapor analyzer using cavity ring-down spectroscopy was employed for trace BTEX analysis, while DI-HS-SPME-GC–MS was utilized for the analysis of all three groups of compounds as well as any other non-target compounds. The DI-SPME method for the extraction of BTEX and PAHs (8) was utilized with minor modifications (Supplementary File 1). Standards and positive controls were included throughout the analyses to ensure optimal analytical results. Chemical analysis of baseline environmental control vs. Surfside building collapse site fluid samples were further compared using a *t*-test assuming unequal variance (9). A *p*-value < 0.05 was considered statistically significant.

### Microbiological analysis

Samples of standing water collected from the Surfside building collapse site, surface wipes obtained from working dogs, and reward items were submitted to Diversigen (Houston, TX, USA) for DNA extraction, amplification, and 16 s rRNA gene amplicon next-generation sequencing. Due to the likely presence of chemical contamination, additional pre-treatment steps were taken for each of the sample types (Supplementary File 2). DNA samples were extracted with MagAttract PowerSoil DNA EP kit (Qiagen) using mechanical-based lysis with garnet beads. The extraction process was automated for high throughput on the Hamilton STARLet. Sequencing libraries were prepared using the AccuPrime High Fidelity kit (Invitrogen) and primers containing single-end barcodes targeting the variable region 4 of the 16S rRNA gene (16S V4; 515F/806R). Amplification was not successful for the wipe samples (Samples 4 and 5) and thus were not included in microbial community analysis. The PCR products were pooled and commercially sequenced using Illumina MiSeq paired end 2 × 250 bp flow cell and the MiSeq Reagent Kit V3 (600 Cycles) (Illumina) by Diversigen.

Cutadapt v2.10 (10) was used to remove adapters from sequencing reads and to truncate reads using the quality trimming algorithm based on a desired Q-score of 30. After truncation, reads of less than 50 base pairs in length were discarded. Quality-controlled reads were then used to generate amplicon sequence variants (ASVs) via dada2 v1.16.0 (11). Paired FASTQ reads were trimmed and filtered to remove reads containing Ns or >2 sequencing errors per read. Forward and reverse reads were merged by overlapping sequences, and chimeras were removed before taxonomic assignment. ASV taxonomy was assigned using the SILVA v.138 database (12) as described by Wang (13). Final ASVs present in the control sample were subtracted from the experimental samples prior to diversity analyses.

Alpha and beta diversity metrics were calculated from the filtered ASV table using RStudio 4.3.2 and the tidyverse packages (14). To generate the alpha diversity metric curves using the vegan package (15), a rarefied community matrix was generated using the rarify function every 10,000 reads up to 57,137 reads (the lowest number of total reads for one of the samples) with a set seed to ensure reproducibility. From these communities, the Chao-1 index, Shannon index, Observed ASV count, and Simpson index were calculated using vegan’s diversity function. Beta diversity metrics were generated using vegan’s vegdist function using the Bray-Curtis diversity index to calculate Non-Metric Dimensional Scaling (NMDS) ecological distances. Principal Coordinate Analysis (PCoA) was assessed using the R package stats’ dist function^1^. Plots were generated using ggplot2 as previously described (16). To determine the statistical power of the Beta analysis, a PERMANOVA test was conducted on the resulting Bray-Curtis distance matrix using the adonis2 function of the vegan package with 999 permutations.

## Results

### Chemical analysis

The AROMA-VOC was used to detect and quantify BTEX in the samples (Table 2). Water from a house gutter (Control 14) contained the highest concentration of toluene at 13.353 ppb, followed by water collected near a construction site (Control 7) and water collected from a driveway (Control 3), at 10.0784 ppb and 10.017 ppb of the environmental baseline control samples. Surfside building collapse site samples contained elevated levels of BTEX in comparison to these environmental baseline control samples (Table 2). Results from the Control and Surfside samples were statistically compared (Table 2). All BTEX compounds were higher in the Surfside samples when compared to controls. Surfside 9 contained the highest concentration of toluene and xylene at 92.391 and 48.908 ppb, respectively.

**Table 2:** Comparison of BTEX concentrations reported by AROMA-VOC from environmental baseline control and Surfside building collapse site aqueous samples.

Environmental baseline controls (South Florida)	Sample	Benzene (ppb)	Toluene (ppb)	Ethylbenzene (ppb)	Xylene (ppb)
Parking lots and driveway	Control 1	0.028	1.305	0.145	0.089
	Control 2	0.317	0.295	0.257	0.192
	Control 3	0.447	10.017	0.356	0.430
	Control 4	0.329	0.826	0.274	0.122
	Control 5	0.287	0.220	0.219	0.079
	Control 6	0.029	0.131	0.047	0.042
	Control 7	0.408	10.784	0.199	0.247
Street	Control 8	0.164	0.909	0.312	0.402
	Control 9	0.270	0.232	0.105	0.068
Backyard and apartment area	Control 10	0.242	2.388	0.331	0.162
	Control 11	0.292	8.980	0.502	0.162
	Control 12	0.359	0.171	0.149	0.014
	Control 13	0.376	4.694	0.171	0.168
	Control 14	0.365	13.353	0.274	0.153
	Control 15	0.266	0.127	0.171	0.108
Average		0.279	3.629	0.234	0.162

Surfside building collapse site	Sample	Benzene (ppb)	Toluene (ppb)	Ethylbenzene (ppb)	Xylene (ppb)
Buildings 1 and 2	Surfside 9	8.588	92.391	0.000	48.908
	Surfside 14	7.072	22.498	1.138	5.122
	Surfside 15	9.711	62.267	1.336	8.619
	Surfside 17	1.034	2.580	0.719	0.699
Decontamination sites	Surfside 11	0.814	3.565	0.386	2.793
	Surfside 12	1.334	1.917	0.000	0.871
	Surfside 13	1.017	3.696	1.123	0.118
Parking lot	Surfside 16	4.307	24.595	2.730	7.365
Average		4.235	26.688	0.929	9.312
*T*-test *p*-value comparing samples to controls		0.010*	0.047*	0.032*	0.078**

*Indicates statistical significance assuming unequal variance; *p* = 0.05.

**When the Surfside 9 sample is removed as an outlier, the difference is statistically significant assuming unequal variance, *p* = 0.18.

DI-SPME-GC–MS was used to detect PAHs and PCBs. Table 3 depicts the overall results of the environmental baseline control and Surfside building collapse site samples. No PCBs were found in either the control or sample groups. Only a few control samples contained PAHs, most notably Control 9, a water sample taken from a street where road work had recently been completed. It is important to note that all of the Surfside samples contained some levels of PAHs.

**Table 3:** Targeted analysis of compounds present in standing liquids from environmental baseline control and Surfside building collapse site samples.

Location	Controls	Polycyclic aromatic hydrocarbons (PAHs)															
		Nap	Acy	Ace	Fle	Ph	An	Fla	Py	B[a]A	Chr	B[a]P	B[b]F	B[k]F	B[ghi]P	Ind	D[a,h]A
Parking lots and driveway	Control 3	X				X		X									
	Control 5	X				X		X	X		X						
	Control 7																
Street	Control 9	X		X	X	X	X	X	X	X	X	X	X	X	X	X	
	Control 10																
Backyard and apartment area	Control 11	X															
	Control 12	X			X	X	X	X	X	X	X	X	X				
	Control 13					X		X	X								
	Control 14					X		X									
	Control 15																
	Control 16	X				X		X	X								

Location	Surfside samples	Nap	Acy	Ace	Fle	Ph	An	Fla	Py	B[a]A	Chr	B[a]P	B[b]F	B[k]F	B[ghi]P	Ind	D[a,h]A
Building 1 and 2	Sample 9	X			X	X	X	X	X	X	X						
	Sample 10	X			X	X	X	X	X	X	X	X	X	X			
	Sample 14	X				X	X	X	X	X	X	X	X	X			
	Sample 15	X				X	X	X									
	Sample 17	X		X	X	X	X	X	X	X	X	X	X	X			
Decontamination area	Sample 11	X		X	X	X	X	X	X	X	X	X	X	X			
	Sample 12	X		X	X	X	X	X	X	X	X	X	X	X	X	X	
	Sample 13	X		X	X	X	X	X	X	X	X	X	X	X	X	X	
Parking lot	Sample 16	X				X	X	X	X	X	X						

*Analysis of Surfside sample 9 & 15 were complicated by large concentrations of unresolved materials. X: Present; X: Present in low abundance below peak area of 10,000; Nap (naphthalene), Acy (acenaphthylene), Ace (acenaphthene), Fle (fluorene), Ph (phenanthrene), An (anthracene), Fla (fluoranthene), Py (pyrene), B[a]A (benz[a]anthracene), Chr (chrysene), B[a]P (benzo[a]pyrene), B[b]F (benzo[b]fluoranthene), B[k]F (benzo[k]fluoranthene), B[ghi]P (benzo[g,h,i]perylene), Ind (indeno(1,2,3-cd)pyrene), D[a,h]A (dibenz[ah]anthracene).

**Figure 2:**
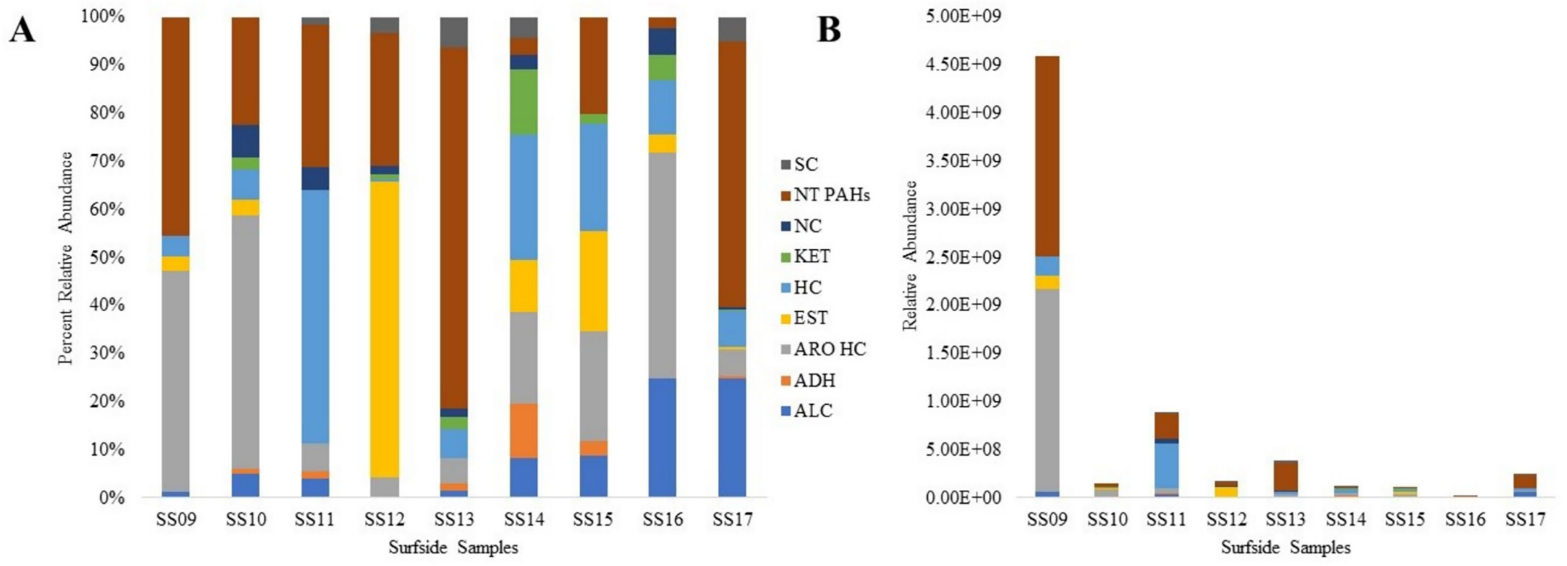
Classification of untargeted compounds found within the Surfside building collapse site fluid samples by **(A)** percent relative abundance of groups and **(B)** overall relative abundance of compounds. ALC (alcohol), ADH (aldehydes), ARO HC (aromatic hydrocarbons), EST (esters), HC (hydrocarbons), KET (ketones), NC (nitrogenous compounds), NT PAHs (non-targeted polycyclic aromatic hydrocarbons), and SC (sulfur containing compounds).

Results from the untargeted analysis are given in Figure 2. Compounds found within Control 16 (Florida International University laboratory tap water) were excluded from the bar plot (Figure 2A). Surfside 9, 10, and 16 were composed mainly of aromatic compounds, while Surfside 13 was composed mainly of PAHs. Only the beginning portion of the chromatogram for Surfside Sample 15 was analyzed due to the unresolved complex materials (UCMs), starting at approximately r_t_ = 15 min (Supplementary File 3). The main ions appearing within the UCM were *m/z* 57, 71, and 85, indicative of hydrocarbons. Figure 2B demonstrates the level of compounds found within each sample, with Surfside 9 containing the most and Surfside 16 containing the least.

HS-SPME-GC–MS was used to detect BTEX, PAHs, and PCBs from the surface wipe and reward item samples. No targeted compounds were found within either the wipe samples or the ball. Fluoranthene and pyrene were both detected on the tug toy (data not shown).

Results of untargeted analysis for the wipe samples are presented in Table 4. Out of the 21 unique identified compounds, five compounds were found within all samples. One compound, tentatively identified as galaxolide, was found in only the pre-decontamination gauze, and two compounds, tentatively identified as Di(2-propylpentyl) ester phthalic acid and pentadecanal, were found in only the post-decontamination gauze. The five tentatively identified compounds found across all samples were benzaldehyde, octanal, 6,10,14-trimethyl-2-pentadecanone, dibutyl phthalate, and isopropyl palmitate. The full list of untargeted compounds can be found in Supplementary File 4.

**Table 4:** Identified compounds in pre- and post-decontamination surface wipes of working dogs not found in the control wipe.

RT (min)	Pre-decontamination		Post-decontamination		
	Canine 1	Canine 2	Canine 3	Canine 4	Canine 5
	Compound	Compound	Compound	Compound	Compound
6.307	Benzaldehyde	Benzaldehyde	Benzaldehyde	Benzaldehyde	Benzaldehyde
7.579	Octanal	Octanal	Octanal	Octanal	Octanal
11.482	Decanal	NA	Decanal	Decanal	Decanal
21.159	6,10,14-trimethyl-2- pentadecanone	6,10,14-trimethyl-2- pentadecanone	6,10,14-trimethyl-2- pentadecanone	6,10,14-trimethyl pentadecanone	6,10,14-trimethyl pentadecanone
21.256	Galaxolide	Galaxolide	Galaxolide	NA	NA
22.375	Dibutyl phthalate	Dibutyl phthalate	Dibutyl phthalate	Dibutyl phthalate	Dibutyl phthalate
22.986	Isopropyl palmitate	Isopropyl palmitate	Isopropyl palmitate	Isopropyl palmitate	Isopropyl palmitate
27.419	NA	NA	NA	Di(2-propylpentyl) ester phthalic acid	Di(2-propylpentyl) ester phthalic acid

### Microbiological analysis

Illumina sequencing of 16S rRNA gene amplicons resulted in a total of 2,121,849 reads from the 15 samples analyzed. Table 5 indicates the number of reads and ASVs remaining after subtracting the corresponding ASVs (*n* = 252) present in the control wipe. Because the tug toy shared 46.3% of its reads with the control wipe (Sample 6), only 57,137 reads and 120 different ASVs were further analyzed.

**Table 5:** Number of 16S rRNA gene V4 region sequencing reads and phylotypes (ASVs).

Sample	Number of reads	Number of ASVs
1- Pre Decon wipe	69,261	1,305
2- Pre Decon wipe	129,236	2,075
3- Pre Decon wipe	156,407	1,528
7- Ball	137,282	1,825
8- Tug toy	57,137	120
9- B1 Puddle 2	129,553	646
10- B1 Puddle 3	213,511	410
11- Decon East	126,729	1,949
12- Decon Walkway	128,611	1,896
13- D2 Puddle	94,531	770
14- B2 Puddle	160,257	645
15- B1 Puddle	127,122	708
16- Parking	116,227	685
17- Center of pile	105,687	589

*Sample 4 and 5 failed amplification.

Sample 6 was ASV’s were subtracted as the control.

**Figure 3:**
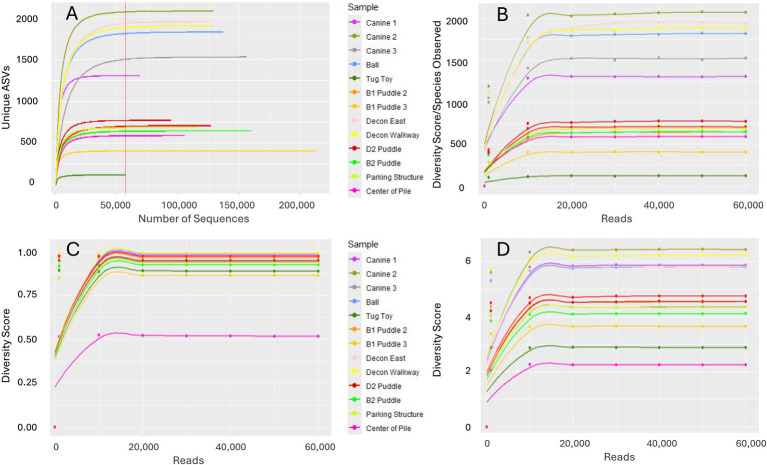
Rarefaction and alpha diversity plots of 16S rRNA sequences. **(A)** Rarefaction curve with vertical line corresponding to 57,137 total sequences analyzed for the tug toy, **(B)** Chao-1 index, **(C)** Simpson index, and **(D)** Shannon index. *Indices in B–D were based on rarefying the data to 57,137 sequences for each sample.

The number of unique ASVs plateaued for all samples during rarefaction analysis indicating further sequencing would not identify additional taxa (Figure 3A).

The Chao-1 index curve indicates that the microbial community of the tug toy contained the fewest species while the microbial community recovered from the pre-decontamination surface wipe of Canine 2 contained the highest level (Figure 3B). These curves also indicated that the communities recovered from the three pre-decontamination surface wipes possessed more species than the puddle, parking structure, and center of pile samples. Overall, the microbial communities recovered from the puddle samples possessed similar species numbers and richness (Figure 3B). Both the Simpson and Shannon indices indicated that the microbial community recovered from the center of the pile was the least diverse of all the samples analyzed (Figures 3C,D).

**Figure 4:**
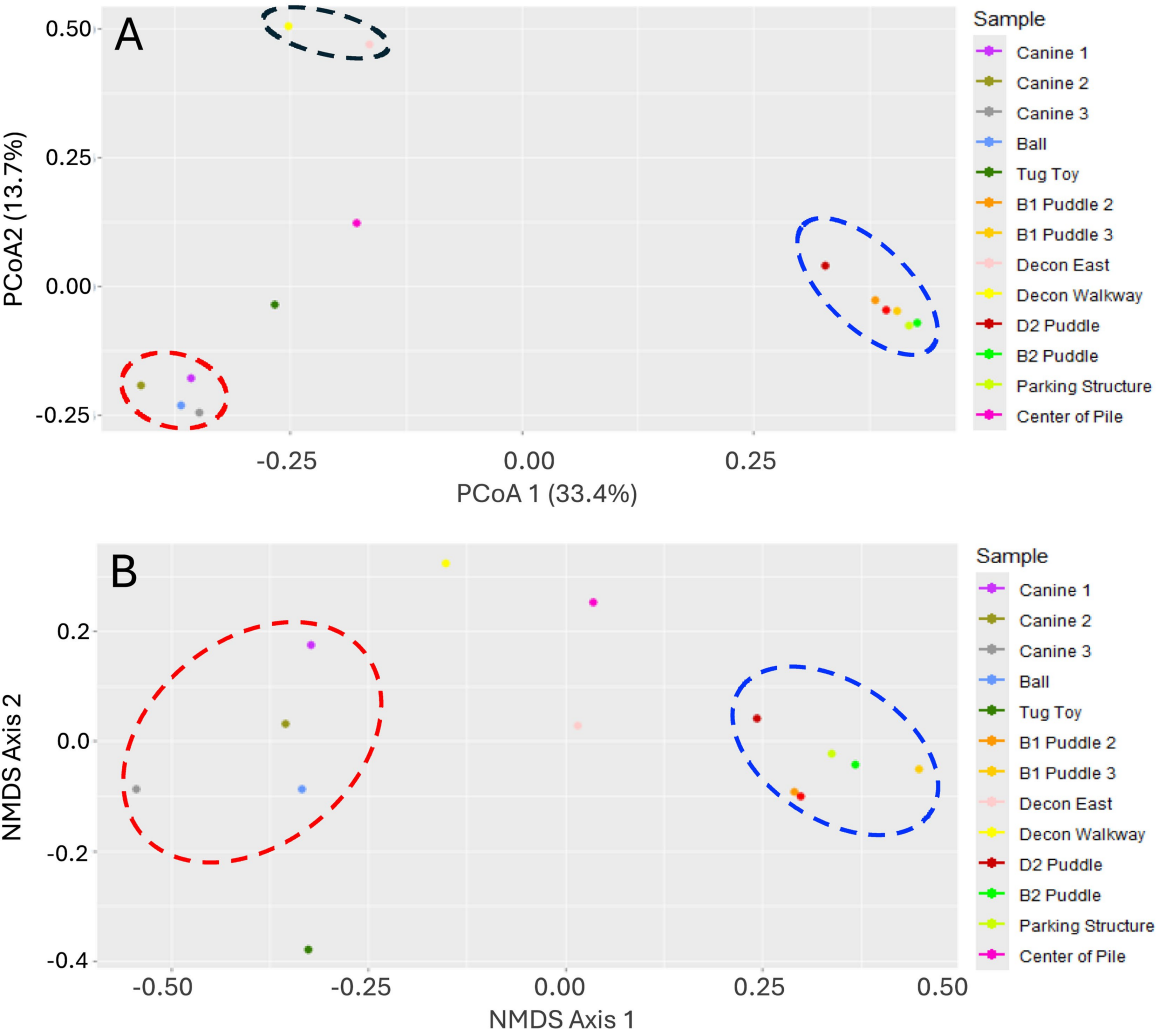
Beta diversity plots of sample microbial communities. **(A)** PCoA and **(B)** NMDS. Dashed ovals highlight samples with similar microbial community dynamics. Adonis analysis indicated significant differences between the microbial communities (R^2^ = 0.24, *p* = 0.003).

Beta diversity analysis of the ASVs detected using Principal Coordinate (PcoA) and Non-Metric Multidimensional Scaling (NMDS) analyses indicated that the microbial communities recovered from the pre-decontamination surface wipe and ball samples were similar to one another and the communities present in the puddle and parking structure samples were similar to one another (Figure 4). The Principal Coordinate Analysis (PCoA) also indicated that the Decon East-11 and Decon Walkway-12 sample communities were more similar than other sample communities (Figure 4A).

**Figure 5:**
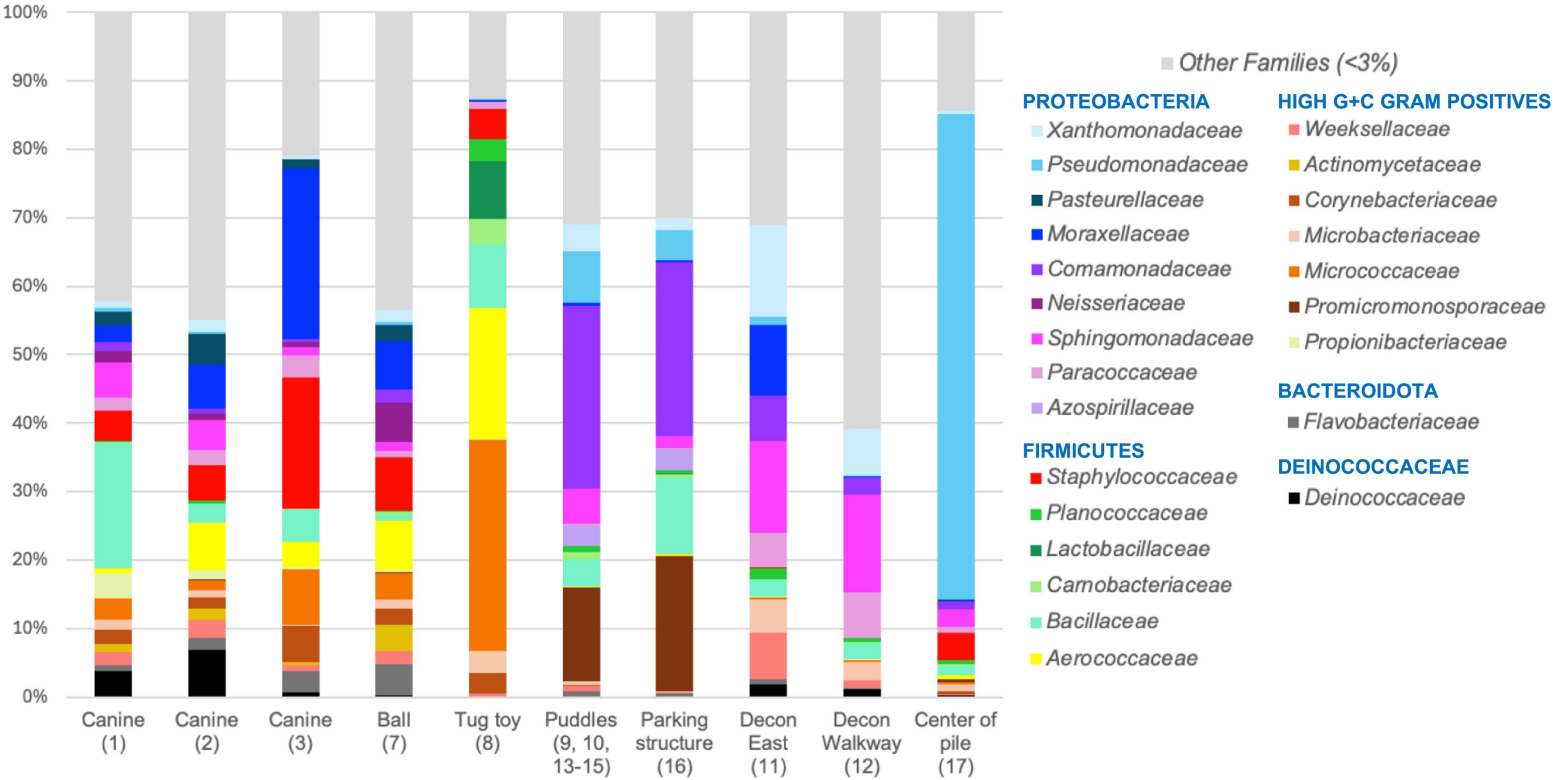
Relative abundance of bacterial families detected in Surfside building collapse site samples. *Taxa detected in puddle samples (Sample ID 9, 10, 13–15) were combined and families detected at less than 3% of total sequencing reads for each sample were assigned as other families.

ASVs were assigned at the family level and relative abundances were determined for each sample. Taxa in families detected at less than 3% abundance were combined into the Other Families category. Because of the high level of beta diversity determined for the microbial communities recovered from all puddle samples, relative family abundances with puddle samples combined are shown in Figure 5.

Microbial data from the pre-decontamination wipes (Canines 1, 2 and 3) as well as the ball reveal similar taxa in different abundances. The six families present with abundances >6% were Moraxellaceae, Staphylococcaceae, Bacillaceae, Aerococcaceae, Micrococcaceae, and Deinococcaceae (Figure 5). Canine 3 possessed the highest percentage of taxa affiliated with the Moraxellaceae and Staphylococcaceae families with 25.02 and 19.11%, respectively. At the genus level, these families were mostly composed of *Psychrobacter*, *Moraxella*, *Macrococcus*, and *Jeotgalicoccus* species that are commonly associated with canine skin, nasal, and oral microbiomes (Supplementary File 5). The main difference between the ball and pre-contamination wipes was an increase in Neisseriaceae, specifically the canine oral genus *Conchiformibius* (5.75% of total reads).

The tug toy contained a predominance of Micrococcaceae and Aerococcaceae with 30.85 and 19.23% of the total ASVs, respectively (Figure 5). The predominant ASVs from these groups belonged to the airborne genera *Glutamicibacter* (21.48% of total reads) and *Aerococcus* (14.43% of total reads; Supplementary File 5). The presence of members of the Planococcaceae, Lactobacillaceae, and Carnobacteriaceae families was also unique to the tug toy, which also explains the dissimilarity of beta diversity analysis (Figure 4). The most abundant genus detected within the families above was *Leuconostoc* (6.31% of total reads), a lactic acid bacterium found in the oral cavity.

Puddle and parking structure samples contained similar taxa with members of the environmentally relevant families Promicromonosporaceae (13.58% of puddles and 19.75% of parking structure) and Comamonadaceae (26.65% of puddles and 25.47% of parking structure) predominating. While a specific genus within Promicromonosporaceae family could not be determined, the genus *Hydrogenphaga* was the dominant Comamonadaceae member detected with 19.89% of the total sequences analyzed in the puddles and 20.65% of the total sequences recovered from the parking structure (Supplementary File 5). While the microbial community detected in standing liquids from the two canine decontamination sites, Decon East and Decon Walkway, possessed members of many of the same families detected in the puddle samples, both decontamination site samples were predominated by diverse metabolizers of the Sphingomonadaceae family (13.44% of ASVs for Decon East and 14.30% of ASVs for Decon Walkway; Figure 5). In contrast to puddle and parking structure samples, decontamination site samples also included taxa from Paracoccaceae, Microbacteriaceae, and Weeksellaceae families which are commonly found in soil and animal samples.

The microbial community present in the liquid collected from the center of the pile possessed the least diversity based on the Simpson and Shannon indices (Figures 3C,D). This community was dominated by members of the Pseudomonadaceae family with 70.95% of the sequencing reads (Figure 5). Within this family, the environmental genus *Pseudomonas* comprised 69.12% of the total sequences (Supplementary File 5). The sample also contained 3.88% of ASVs belonging to the Staphylococcaceae family. Common genera such as *Macrococcus* and *Staphylococcus* were detected at 2.03 and 1.75% of the total sequences.

## Discussion

In a cross-sectional evaluation of environmental contaminants present at the Surfside building collapse site conducted during ongoing US&R operations involving working dogs, chemical and microbiological contaminants persisted nearly 3 weeks after the initial event. Elevated levels of benzene, toluene, ethylbenzene, and xylenes (BTEX) and polycyclic aromatic hydrocarbons (PAH) were identified in chemical analyses, while members of the Pseudomonadaceae and Staphylococcaceae family were detected using 16 s next-generation sequencing. These data are the first to identify chemical and microbial contaminants US&R working dogs may come in direct contact with during search, rescue and recovery operations after a large building collapse.

### Chemical contamination at the Surfside building collapse site

In this study, the use of AROMA-VOC and GC–MS can be viewed as complementary, with AROMA-VOC facilitating analysis of BTEX (highly volatile compounds) and GC–MS facilitating the analysis of PAHs and PCBs (volatile and semi-volatile compounds). The results from the AROMA-VOC showed that all aqueous samples had a measurable concentration of BTEX. Studies conducted on rainwater (17), groundwater (18) and storm water (19) have demonstrated low levels of these compounds due to the widespread use of products such as gasoline, which contain BTEX. Although some of the levels of BTEX in the Surfside building collapse site samples were under the U. S. Environmental Protection Agency’s allowed limit of BTEX in public drinking water (5 ppb for benzene, 1 ppm for toluene, 0.7 ppm for ethylbenzene, and 10 ppm for xylene), Samples 9, 14, and 15 far exceeded the allowed limit of benzene (43).

Aqueous samples gathered from the Surfside building collapse site differed greatly in overall chemical composition. It is noteworthy that no PCBs were identified in any of the samples, likely due to legal prohibitions against the use of PCBs in building materials beginning in the early 1970s. The Champlain Towers were built in 1981 (20). In contrast, elevated levels of PAHs were found in almost all of the samples in varying quantities.

Similar to the AROMA-VOC targeted analysis, type and amount of targeted compounds assessed using GC–MS varied widely across all Surfside samples. Only a few of the control samples contained these targeted compounds, most notably Control 9 (Miami street with recent road repair). Asphalt is a semi-solid form of petroleum and is a typical material used in road repairs. Other work has reported the release of PAHs during asphalt operations (21) which likely explains the presence of PAHs in this versus other control samples. The presence of PAHs in the Surfside samples may be attributed to fires that occurred during US&R operations.

Untargeted analysis of aqueous samples also revealed differing compounds. The main classes of compounds found throughout all the samples were aromatic hydrocarbons, hydrocarbons, and non-targeted PAHs. Many of the identified compounds were comparable to those found in prior studies that focused on the 9–11 attacks at the World Trade Center (22) and the combustion products of household and building materials (44).

HS-SPME and GC–MS were used to analyze both surface wipe and reward item samples, and only the tug toy contained target compounds. The PAHs identified from the tug toy were fluoranthene and pyrene. Fluoranthene and pyrene are compounds found naturally in crude oil, fossil fuels, gasoline exhaust, and smoke from cigarettes and burning wood. These compounds can also be found in food products, especially grilled foods such as hamburgers and hot dogs, as well as dyes, plastics, and pesticides. Studies have yet to be conducted on the health effects related to short term exposure to these compounds. However, animal studies have shown that long term exposure can lead to kidney disease (23).

Five compounds were identified via untargeted analysis across all wipe samples (both pre and post decontamination) (Table 4). One compound, 6,10,14-trimethyl-2-pentadecanone, is a ketone which is produced by pyrolysis of plant constituents and degradation of aldehydes and alcohols by bacteria. Residual plant tissue can be the source of this (24). Dibutyl phthalate is used in making flexible plastics that are found in many consumer products. The EPA reports that dibutyl phthalate may have relatively low acute and chronic toxicity. Animal studies have reported developmental and reproductive effects from oral exposure. Dibutyl phthalate can be found in fish and other seafood (25). Finally, isopropyl palmitate is a fatty acid ester that has a role as a human metabolite. It is a natural product found in *Camellia sinesis* (tea), *Solanum tuberosum* (potato), and other organisms. Isopropyl palmitate is used in cosmetic and personal care products (26).

When comparing the two reward items, heneicosane was the only compound found in common. Heneicosane is a straight chained alkane that is a component in crude oil, a plant metabolite in fruits such as elderberries and lemon balms, and is pheromone used by termites (46). Both reward items contain different hydrocarbons, which could have been deposited on the items due to residue from the rubble fires at the Surfside building collapse site. Overall, the compounds found in both surface wipe and reward item samples may have come from household items, chemicals, or biological materials that may have been buried, broken, or crushed under the rubble.

Chemicals identified on working dogs and reward toys post-decontamination are particularly noteworthy for potential health effects. Unfortunately, veterinary medical literature contains little information regarding the impact of these environmental contaminants on the health of dogs.

### Microbial composition of samples collected at the Surfside building collapse site

Microbial community analysis of surface wipe samples collected from working dogs and fluid samples from the Surfside building collapse site indicated a low risk of pathogen exposure from sewage sources. No families of coliform origin were detected at an appreciable level (Figure 5). A predominance of members from the Pseudomonadaceae family and presence of members from the Staphylococcaceae family at the center of the pile is interesting as certain pathogenic species from either family could pose a health threat to canines and humans alike.

Despite additional precautionary steps taken prior to extraction (Supplementary File 2), taxa associated with pathogenesis or sewage detected in the samples surrounding the collapse could have been negatively impacted by the presence of BTEX and PAHs, which is consistent with the findings from the chemical analysis. Promicromonosporaceae, Comamonadaceae (genus *Hydrogenphaga*), and Sphingomonadaceae members that dominated the puddle, parking structure, and decontamination site (Decon East and Decon walkway) samples (Figure 5; Supplementary File 5) have been associated with organic polymer degradation and BTEX pollutants (27–32). Additionally, the time elapsed between the Surfside building collapse and sample collection as well as persistent rainfall may have resulted in fluctuations in both microbial and chemical compositions in the environment during US&R operations due to the diluting effect of rainfall.

Overall, the microbial communities present on the surface wipes were distinctly different from the microbial communities present in fluid samples collected from standing water in the search area. This was likely a consequence of the wipes being applied to the back of the canines versus their paws. The pre-decontamination wipes and ball contained DNA traditionally associated with canine microbiota such as the Moraxellaceae genera *Psychrobacter* (33, 34) and *Moraxella* (35) as well as the Staphylococcaceae genera *Macrococcus* and *Jeotgalicoccus* (Figure 5; Supplementary File 5). *Macrococcus* species have been detected as part of the non-allergic healthy skin microbiota of German shepherd dogs (36) and *Jeotgalicoccus* species have been detected as normal canine ear and gut microbiota (37, 38).

Microbiota present on the ball notably included members of the Neisseriaceae family, which are frequently commensal and colonize the mucosa of the oropharynx of animals and humans. *Conchiformibius*, the dominant genus detected (Supplementary File 5), is a part of the canine oral microbiome (35). Although taxa identified on the Tug toy were distinct from all other samples (Figure 5), members of Planococcaceae, Lactobacillaceae, and Carnobacteriaceae within the Firmicutes have been previously associated with canine gut and oral microbiomes (39–41).

Future studies should include evaluation of microbial and chemical data from the paws of working dogs pre- and post-decontamination. This data may better represent the contamination risk from standing water and walking/climbing through a contaminated site. Previous studies (42) have reported that handlers spend more time cleaning the back of their working dog than the paws, even when they know the paws have been contaminated. Dogs may ingest contaminants during self-grooming, therefore data on the microbial and chemical contamination of paws is essential to maintaining working dog health.

### Practical implications for working dog decontamination

Decontamination of working dogs operating in a disaster setting can be highly variable. US&R canine teams deployed to the Surfside building collapse utilized both full bath and wipe down procedures. Although canine decontamination is an established priority, it may not be executed consistently due to lack of resources. Contaminant form (liquid, solid, or aerosolized particulates) may also influence the effectiveness of the decontamination method employed. Similarly, degree of contamination must also be considered. Different strategies may be necessary to effectively remove contaminants after surface coat exposure vs. whole body immersion in water hazards. Likewise, adherence of solid contaminants to the coat may require other methods. Standards for a decontamination decision matrix should be incorporated into canine handler training and best practices.

Effective working dog decontamination is critical not only to protecting the health of the dog but that of the handler and other personnel. While the long-term consequences of exposure to working dogs contaminated with chemical and microbial contaminants on human health is not known, cross-contamination is plausible. This underscores the importance of comprehensive decontamination strategies that encompass working dog and handlers alike in disaster settings.

### Limitations

As this study was conducted using samples collected from working dogs as well as several locations within a contaminated environment at a single point in time after a building collapse, sampling bias is a significant limitation. Sample collection did not occur until nearly 3 weeks after the event; therefore, chemical and microbiological contaminant profiles identified may not reflect threats present immediately after the collapse. Contaminant dilution by rainfall or other water sources; redistribution by ventilation, wind, or recovery operations (e.g., delayering of rubble and other debris); and/or degradation via sunlight or dessication over time could have altered these profiles. Nevertheless, these findings retain relevance as search and recovery operations after large disasters may be prolonged and prolonged or repeated exposure is often necessary during operations. Additional follow on analyses should be completed in order to characterize the full spectrum of hazards encountered by deployed dogs. These should include liquid chromatography and inductively coupled plasma mass spectrometry for the analysis of larger molecules and metals, respectively. Species-level characterization of microbial communities should be pursued to assess for the presence of microorganisms with significant pathogenic potential (e.g., *Pseudomonas aeruginosa*, *Staphylococcus aureus*) in humans and canines alike.

## Conclusion

Working dogs deployed to large building collapse sites risk exposure to chemical and microbiological contaminants. BTEX, PAHs, and microorganisms from the Pseudomonadaceae and Staphylococcaceae families contaminated standing water at the Surfside building collapse site weeks after the event, posing a potential threat to canine and human health. Future disaster responses should include structured environmental hazard assessments to better inform guidance and best practices surrounding working dog decontamination.

## Data Availability

The datasets presented in this study can be found in online repositories. The ASV sequencing data were deposited in the NCBI Sequence Read Archive under accession number PRJNA1404321.

## Footnotes

1
https://www.R-project.org
