# Supplementary material for: An analysis of working dog exposure to environmental contaminants at the Surfside building collapse
Source: Front Vet Sci. 2026 Mar 2;13:1725234. doi: 10.3389/fvets.2026.1725234 (PMC12989390; doi:10.3389/fvets.2026.1725234)
Supplement: Supplementary file 1 [file Data_Sheet_1.docx]

**Supplemental File 1**. Modification of DI-SPME extraction method developed by J. Bianchin et al. (2012) for extraction of BTEX and PAHs.

A method developed by J. Bianchin et al. (2012) for the extraction of BTEX and PAHs was used for this analysis with minor modifications, summarized in Table S1. A positive control consisting of 10 mL of tap water containing 37.5 ppb of BTEX and PCBs and 25 ppb of PAHs was run using the developed extraction method. Next, 10 mL of thawed samples were distributed into a 20 mL VOA vial. The samples were stirred for an hour for homogenization. The stir bar was removed, and samples were heated to 80 ℃ in an aluminum heating block and extracted using DI-SPME for 48 minutes. Samples were then removed from the heating block and placed into a water bath at approximately 20 ℃ and extracted again using HS-SPME for 32 min.^10^ HS-SPME was carried out for the wipe and rewards samples.

Samples were analyzed using an Agilent 8890 GC and 5977B MSD equipped with an HP5-MS (Agilent 15 m x 0.25 mm I.D. x 0.25 μm). The method parameters are in Table S2. After extraction of the sample, SPME fibers were desorbed in the inlet at 250 °C for 4 minutes. Compounds were tentatively identified by comparison matching to those compounds in the NIST mass spectra library (version NIST20). Targeted compounds were identified using reference materials of BTEX standard (SPEX CertiPrep), 550-A PAH standard (SPEX CertiPrep), and PCBs standard (Aroclor ®).

Table S1:DI-HS-SPME parameters for liquid samples (Bianchin et al., 2012).

|  | **DI** | **HS** |
| --- | --- | --- |
| **Equilibration** | 0 min | 0 min |
| **Extraction** | 48 min | 32 min |
| **Temperature** | 80 °C | 10 °C |
| **SPME Fiber Type** | 100 μm PDMS | |

Table S2: GC-MS parameters used for all samples.

| Method Parameter |  |
| --- | --- |
| Inlet mode | Temperature: 250 °C  10:1 split  1 mL/min flow rate |
| Oven program | 35 °C, hold 2 min  35-60 °C at 5 °C/min  60-110 °C at 10 °C/min  110-130 °C at 5 °C/min  130-300 °C at 10 °C/min, 2 min hold |
| Mass Spec Parameters | Transfer Line at 280 °C  Mass Range (m/z) 50 to 550 |
| Column Type | HP5-MS 15 m x 0.25 mm I.D. x 0.25 μm |

**Supplemental File 2.** Additional pre-treatment steps for fluid samples with likely presence of chemical contamination prior to DNA extraction, amplification, and 16s next-generation sequencing.

**Reward items**

Sterile BD EZ Culture swabs (Cat. No 220149) were used to thoroughly swab reward items. Prior to swabbing, each swab was immersed into 1x PBS (pre-wetting solution). Samples were swabbed vigorously for at least 60s or until the entire surface area of the reward item was thoroughly swabbed. After swabbing, the swab was clipped to a centrifuge tube. Lysis buffer compatible with Diversigen’s Low Biomass Extraction method (1mL 0.5X OR-29) was added into the tube (800ul or as much as needed to obtain 800ul removeable volume). Contents were vortexed and swab and buffer were treated with the standard 20mg/ml Proteinase K treatment and 2-hour dry incubation at 50℃.

**Decontamination wipes**

Two sterile, 5mm steel beads (Qiagen) were added to each tube containing the portioned wet wipe using sterile forceps. Lysis buffer (1 ml) compatible with Diversigen’s Low Biomass Extraction method (0.5X OR-29) was added to each tube and the tube was shaken 30x and then vortexed at max speed for 30s each. Liquid contents were transferred to a centrifuge tube. Contents were treated with 20mg/ml Proteinase K treatment and allowed 2-hour dry incubation at 50℃.

**Standing liquids**

A 25mm 0.2micron filter was added to a demountable Luer lock filter holder using sterilized forceps. Liquid sample contents were transferred to a new 10ml sterile syringe. The filter was attached to the filter holder containing the 0.2micron filter and the sample pushed through the filter. The filter was removed using sterilized forceps and placed into a 15ml filter tube containing 1mL 0.5X OR-29. Two sterile, 5mm steel beads were added, and the sample was shaken 30x and then vortexed at max speed for 30s each. Contents were treated with 20mg/ml Proteinase K treatment and allowed a 2-hour dry incubation at 50℃.

**Supplemental File 3.** Chromatogram of Unresolved Compound Materials (UCM) present in Surfside building collapse site fluid samples.

Figure S1: All total ion chromatograms (TIC) from aqueous samples collected at the Surfside building collapse site.


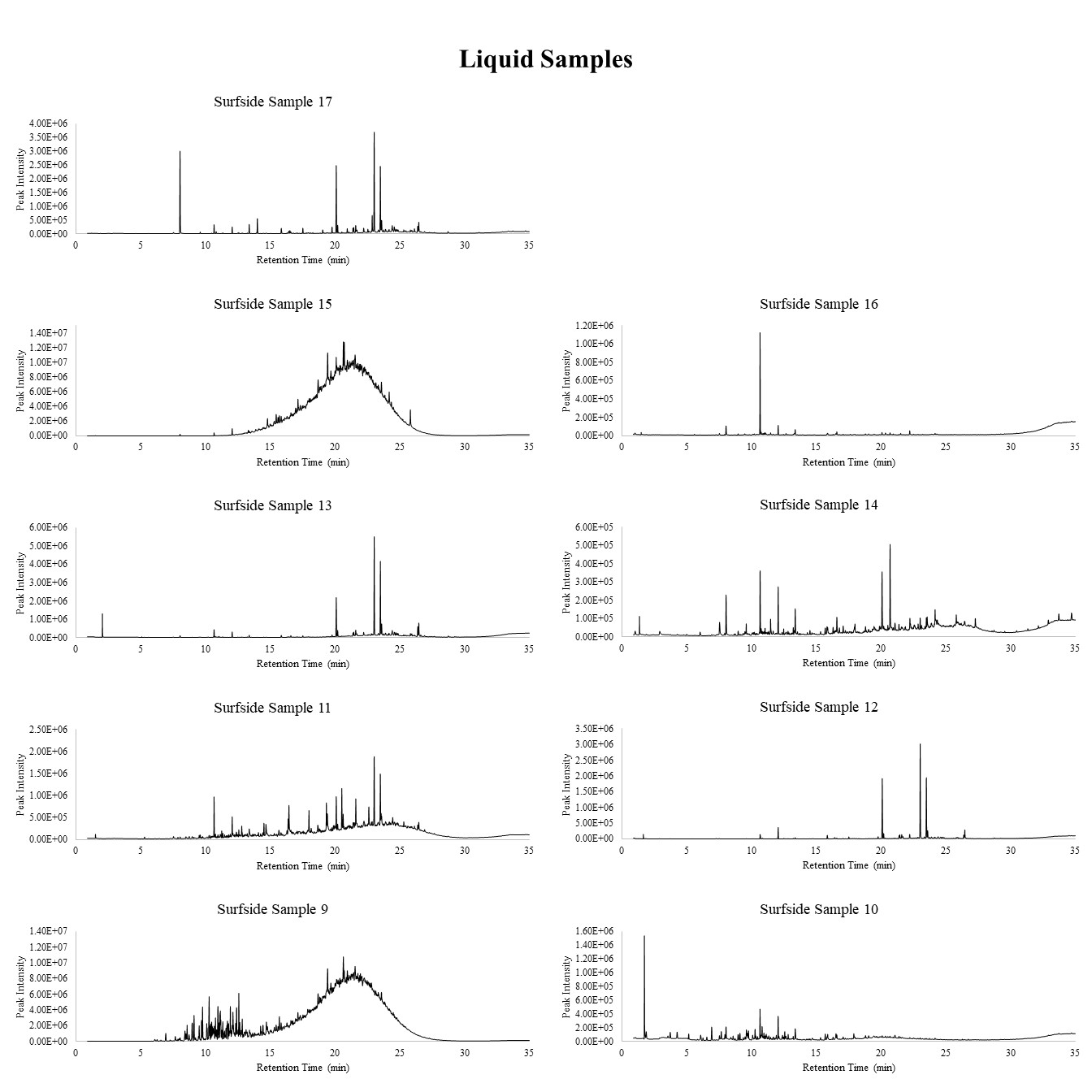


**Supplemental File 4.** Untargeted compounds identified from the headspace of reward items.

Table S2: Identified compounds from the headspace of the reward toys that were not found within the control samples.

| **RT (min)** | **Compounds Identified in Kong** | **RT (min)** | **Compounds Identified in Tug Toy** |
| --- | --- | --- | --- |
| 1.951 | 1,6-dideoxy-1-mannitol | 6.449 | Dimethyl trisulfide |
| 3.176 | 2-ethyl-hexanal | 7.835 | 2,6-dimethyl nonane |
| 3.960 | 1(1-cyclohexen-1-yl) ethanone | 8.213 | 2-ethyl-1-hexanol |
| 5.465 | 1-methyl cycloheptanol | 9.240 | 1,1,3,4-tetramethyl cyclopentane |
| 7.138 | Phenol | 12.043 | 4,6-dimethyl dodecane |
| 8.253 | 2,3,6,7-tetramethyl octane | 12.193 | 1,3-bis(1,1-dimethylethyl) benzene |
| 8.364 | Dihydro-5-methyl-5-vinyl-2(3H)-furanone | 13.234 | 2-methyl-1-decanol |
| 10.124 | N,N-dimethylvaleramide | 19.572 | 2,6,10,14-tetramethyl pentadecane |
| 10.857 | Benzoic acid | 20.770 | 2,6,10,14-tetramethyl hexadecane |
| 11.345 | Dodecane | 21.728 | Nonadecane |
| 11.630 | Benzothiazole | 21.826 | (1-methyldodecyl) benzene |
| 12.860 | 2-(1,1-dimethylethyl) cyclohexanol | 22.732 | Eicosane |
| 12.980 | Tridecane | 23.683 | Heneicosane |
| 13.737 | 1(3H) Isobenzofuranone |  |  |
| 13.931 | Triacetin |  |  |
| 14.059 | Dihydro-5-pentyl-2(3H) furanone |  |  |
| 14.725 | Vanillin |  |  |
| 16.331 | 4-methyl-1,1'-biphenyl |  |  |
| 17.879 | Diethyltoluamide |  |  |
| 18.156 | 2,2,4-trimethyl-1,3-pentanediol diisobutyrate |  |  |
| 18.448 | 2-methyl benzenesulfonamide |  |  |
| 18.987 | 4-methyl benzenesulfonamide |  |  |
| 20.245 | Benzyl benzoate |  |  |
| 20.949 | Dicyclohexyl disulphide |  |  |
| 21.399 | N-cyclohexyl benzamide |  |  |
| 22.230 | N-cyclohexyl phthalimide |  |  |
| 23.511 | 6-methoxy thyml tiglate |  |  |
| 23.683 | Heneicosane |  |  |
| 23.945 | 2,4-dimethyl diphenyl sulfone |  |  |
| 26.701 | Phenothiazine |  |  |

**Supplemental File 5.** Abundance of microbiological ASV’s from all samples.

*Table S.5: Relative abundances and taxonomic assignment of ASVs occurring in at least one sample at >3%.*

| **Family^a^** | **Genus^b^** | **% of abundance of ASVs per sample** | | | | | | | | | |  |
| --- | --- | --- | --- | --- | --- | --- | --- | --- | --- | --- | --- | --- |
|  |  |  |  |  |  |  |  |  |  |  |  | |
|  |  | Canine  1 | Canine  2 | Canine  3 | Ball  7 | Toy  8 | Puddles  8,9,13,15 | Decon  East 11 | Decon  Walk 12 | Parking  16 | Center  Pile 17 | |
| **Xanthomonadaceae** |  | **1.15** | **1.73** | **0.56** | **1.78** | **0.01** | **4.01** | **13.33** | **6.83** | **1.79** | **0.40** | |
|  | *Lysobacter* | 0.40 | 0.51 | 0.09 | 0.14 | - | 0.01 | 6.99 | 3.16 | 0.06 | 0.04 | |
| **Pseudomonadaceae** | *Pseudomonas* | **0.52** | **0.42** | **0.07** | **0.49** | **0.21** | **7.43** | **1.19** | **0.17** | **4.47** | **70.95** | |
| **Pasteurellaceae** |  | **1.95** | **4.42** | **1.26** | **2.25** | **-** | **-** | **-** | **-** | **-** | **0.11** | |
| **Moraxellaceae** |  | **2.53** | **6.36** | **25.02** | **7.22** | **0.26** | **0.50** | **10.31** | **0.13** | **0.24** | **0.19** | |
|  | *Psychrobacter* | 0.34 | 1.58 | 19.12 | 0.31 | 0.20 | - | - | - | - | - | |
|  | *Moraxella* | 0.14 | 0.80 | 5.25 | 6.29 | - | - | - | - | - | - | |
| **Comamonadaceae** |  | **1.21** | **0.82** | **0.46** | **1.79** | **0.03** | **26.65** | **6.74** | **2.49** | **25.47** | **1.20** | |
|  | *Hydrogenphaga* | 0.03 | 0.20 | 0.01 | 0.11 | - | 19.89 | 2.55 | 0.76 | 20.65 | 0.18 | |
| **Neisseriaceae** | *Conchiformibius* | **1.76** | **0.94** | **0.62** | **5.75** | **-** | **-** | **-** | **-** | **-** | **-** | |
| **Sphingomonadaceae** |  | **5.02** | **4.34** | **1.27** | **1.38** | **-** | **5.13** | **13.44** | **14.30** | **1.75** | **2.48** | |
|  | *Sphingomonas* | 2.13 | 1.85 | 0.45 | 0.59 | - | 0.23 | 3.32 | 6.84 | 0.14 | 0.55 | |
| **Paracoccaceae** |  | **1.93** | **2.23** | **3.21** | **0.92** | **1.03** | **0.32** | **4.88** | **6.57** | **0.07** | **0.88** | |
| **Azospirillaceae** |  | **-** | **0.04** | **-** | **-** | **-** | **3.04** | **0.10** | **0.10** | **3.09** | **0.06** | |
| **Staphylococcaceae** |  | **4.44** | **5.17** | **19.11** | **7.74** | **4.41** | **-** | **0.06** | **0.02** | **-** | **3.88** | |
|  | *Jeotglicoccus* | - | 0.15 | 7.68 | 0.26 | 1.76 | - | - | - | - | 0.62 | |
|  | *Macrococcus* | 0.46 | 3.71 | 8.98 | - | 0.79 | - | - | - | - | 0.87 | |
|  | *Salinicoccus* | - | - | - | 5.80 | 0.27 | - | - | - | - | 0.38 | |
|  | *Staphylococcus* | 3.71 | 1.14 | 2.01 | 1.17 | 0.36 | - | - | - | - | 0.64 | |
| **Planococcaceae** |  | **0.21** | **0.33** | **0.09** | **0.18** | **3.18** | **0.76** | **1.74** | **0.52** | **0.55** | **0.65** | |
| **Lactobacillaceae** |  | **-** | **0.03** | **0.02** | **-** | **8.40** | **-** | **-** | **-** | **0.50** | **-** | |
|  | *Leuconostoc* | - | - | - | - | 6.31 | - | - | - | 0.05 | - | |
| **Carnobacteriaceae** |  | **0.21** | **0.04** | **0.05** | **0.53** | **3.76** | **1.19** | **-** | **0.15** | **0.42** | **0.23** | |
| **Bacillaceae** |  | **18.24** | **2.80** | **4.76** | **0.83** | **9.29** | **3.93** | **2.48** | **2.30** | **11.24** | **1.33** | |
|  | *Anaerobacillus* | 3.22 | 0.35 | 0.79 | - | - | 0.05 | - | - | - | 0.30 | |
| **Aerococcaceae** |  | **0.78** | **6.88** | **3.76** | **7.20** | **19.23** | **0.15** | **0.02** | **-** | **0.26** | **0.66** | |
|  | *Aerococcus* | 0.15 | 6.09 | 3.28 | 6.57 | 14.43 | 0.15 | 0.02 | - | 0.26 | 0.66 | |
| **Propionibacteriaceae** |  | **3.55** | **1.44** | **0.19** | **0.24** | **-** | **-** | **0.10** | **0.25** | **-** | **0.04** | |
|  | *Cutibacterium* | 3.25 | 1.08 | 0.16 | 0.04 | - | - | - | - | - | - | |
| **Promicromonosporaceae** |  | **0.04** | **0.03** | **0.02** | **0.21** | **0.03** | **13.58** | **0.04** | **-** | **19.75** | **0.42** | |
| **Micrococcaceae** |  | **3.11** | **1.57** | **8.15** | **3.90** | **30.85** | **0.05** | **0.23** | **0.23** | **-** | **0.23** | |
|  | *Glutamicibacter* | 0.07 | 0.20 | 0.02 | 1.07 | 21.48 | - | .04 | - | - | - | |
|  | *Micrococcus* | 0.04 | 0.21 | 7.70 | 2.09 | 0.07 | - | - | 0.06 | - | 0.08 | |
| **Microbacteriaceae** |  | **1.42** | **1.01** | **0.19** | **1.29** | **3.33** | **0.68** | **4.90** | **2.62** | **0.14** | **1.13** | |
| **Corynebacteriaceae** | *Corynebacterium* | **2.20** | **1.56** | **5.32** | **2.29** | **2.87** | **0.06** | **-** | **-** | **-** | **0.32** | |
| **Actinomycetaceae** |  | **1.08** | **1.61** | **0.38** | **3.80** | **-** | **-** | **-** | **-** | **-** | **0.08** | |
| **Weeksellaceae** |  | **2.00** | **2.78** | **0.93** | **1.93** | **0.45** | **0.75** | **6.77** | **1.20** | **0.28** | **0.11** | |
| **Flavobacteriaceae** |  | **0.89** | **1.74** | **3.05** | **4.59** | **0.06** | **0.80** | **0.77** | **0.08** | **0.44** | **0.03** | |
| **Deinococcaceae** | *Deinococcus* | **3.70** | **6.82** | **0.66** | **0.25** | **-** | **-** | **1.78** | **1.17** | **-** | **0.20** | |
| **Other families (<3%)** |  | **42.08** | **44.91** | **20.84** | **43.43** | **12.59** | **30.91** | **31.09** | **60.84** | **29.98** | **14.41** | |

^a^Abundance of ASVs as assigned to each family presented in bold.

^b^Not all ASVs were resolved at the genus level for every family and some were detected <3%. Relative abundance for genus is based on total ASVs, not percentage of assigned family.
